# Cyclophilin A Inhibits Human Respiratory Syncytial Virus (RSV) Replication by Binding to RSV-N through Its PPIase Activity

**DOI:** 10.1128/JVI.00563-21

**Published:** 2021-07-12

**Authors:** Wenzhang Liang, Yue Zhang, Miao Li, Fadhl Al-Shaebi, Jian Li, Jing Zhang, Lin Wei

**Affiliations:** a Department of Immunology, Hebei Medical University, Shijiazhuang, Hebei, China; b Key Laboratory of Immune Mechanism and Intervention on Serious Disease in Hebei Province, Shijiazhuang, Hebei, China; c Department of Pathogen Biology, Hebei Medical University, Shijiazhuang, Hebei, China; Instituto de Biotecnologia/UNAM

**Keywords:** cypA, PPIase, RSV-N, CSA, viral replication

## Abstract

Human respiratory syncytial virus (hRSV) is the most common pathogen which causes acute lower respiratory infection (ALRI) in infants. Recently, virus-host interaction has become a hot spot of virus-related research, and it needs to be further elaborated for RSV infection. In this study, we found that RSV infection significantly increased the expression of cyclophilin A (cypA) in clinical patients, mice, and epithelial cells. Therefore, we evaluated the function of cypA in RSV replication and demonstrated that virus proliferation was accelerated in cypA knockdown host cells but restrained in cypA-overexpressing host cells. Furthermore, we proved that cypA limited RSV replication depending on its PPIase activity. Moreover, we performed liquid chromatography-mass spectrometry, and the results showed that cypA could interact with several viral proteins, such as RSV-N, RSV-P, and RSV-M2-1. Finally, the interaction between cypA and RSV-N was certified by coimmunoprecipitation and immunofluorescence. Those results provided strong evidence that cypA may play an inhibitory role in RSV replication through interaction with RSV-N via its PPIase activity.

**IMPORTANCE** RSV-N, packed in the viral genome to form the ribonucleoprotein (RNP) complex, which is recognized by the RSV RNA-dependent RNA polymerase (RdRp) complex to initiate viral replication and transcription, plays an indispensable role in the viral biosynthesis process. cypA, binding to RSV-N, may impair this function by weakening the interaction between RSV-N and RSV-P, thus leading to decreased viral production. Our research provides novel insight into cypA antiviral function, including binding to viral capsid protein to inhibit viral replication, which may be helpful for new antiviral drug exploration.

## INTRODUCTION

Human respiratory syncytial virus (hRSV), which is related to about 22% of severe acute lower respiratory tract infections (ALRTI) worldwide (1), mainly infects children under 5 years old, the elderly, and persons who use immunosuppressive agents (2). The condition is more severe among infants with bronchial dysplasia (BPD) (3) or congenital heart disease (CHD) (4). However, there are still no approved vaccines or specific therapeutic drugs to treat RSV infection (5). The usual strategies for RSV infection are mainly dependent on supportive therapies, such as oxygen inhalation and sputum aspiration.

Cyclophilin A (cypA), which is one of the most important members of the immunophilin family and is widely distributed in the cytoplasm and nucleus in eukaryotic and prokaryotic cells, has a hydrophobic pocket formed by eight β folds in the molecule, which has a special functional proline *cis*-*trans*-isomerase activity (PPIase activity) (6). Studies have illustrated that cypA plays an important role in multiple physiological functions, such as protein folding, transport, and T-lymphocyte activation (7). In addition, cypA is an intracellular receptor of cyclosporine (CSA), an immunosuppressive agent widely used in clinical practice (8). In recent years, several studies have shown that cypA directly or indirectly influences the proliferation of viruses by binding with structural or nonstructural proteins of viruses. For example, cypA could combine with Pr55gag, the Gag polyprotein of human immunodeficiency virus type 1 (HIV-1), through its PPIase activity to promote viral replication (8, 9); PPIase activity of cypA inhibitors such as CSA, Debio 025, and NIM811 could block the interaction of cypA and hepatitis C virus (HCV) NS5A/5B protein complex, thus inhibiting viral replication (10). Unlike for HIV and HCV, cypA could bind to M1 of influenza A virus (IAV) and inhibit virus replication, but this inhibition has nothing to do with its PPIase activity (11). In addition, cypA affects the replication of enterovirus 71 (EV71) (12), Epstein-Barr virus (EBV) (13), and coronaviruses (severe acute respiratory syndrome coronavirus [SARS-CoV] and human CoV 229E) (14–17). In view of the role of cypA in the replication of many viruses (18), cypA can be used as a potential target to treat viral infections.

RSV belongs to the genus *Pneumovirus* in the *Pneumoviridae* family in the *Mononegavirales* order (19); it has a genome of about 15 kb, encoding 10 proteins, including 3 envelope proteins (F, G, and SH), 3 capsid proteins (N, P, and L), M1 protein, M2 protein, and 2 nonstructural proteins (NS1 and NS2). The capsid proteins N, P, and L are the main components of the viral RNA-dependent RNA polymerase (RdRp) complex (19), among which the nucleocapsid protein N wraps the viral genome to form a left-handed helical ribonucleoprotein (RNP) complex (20–22), which is the template for viral replication and transcription; L protein is one of the most important components of RdRp, which has three conserved domains, namely, (i) the RNA-dependent RNA polymerase domain, (ii) the polyribonucleotidyl transferase (PRNTase) domain, and (iii) the methyltransferase (MTase) domain (23). P protein is a major cofactor of L protein, which plays an auxiliary role in recognizing RdRp of the RNP complex (24). When the viral genome is transcribed, the fourth factor of virus protein M2-1, called transcription anti-termination factor, is needed, and its function also depends on P phosphorylation (25). Therefore, the integrity of the complex of L-RNP, L-P, RNP-P, and P–M2-1, which are most important critical components in viral replication that could be taken as targets for designing drugs to treat RSV infection (26, 27), has become a hot spot in the research of direct-acting antiviral agents (DAAs) against RSV infection.

In our previous transcriptome sequencing experiments with RSV-infected cells, high expression of cypA attracted our attention, and we speculated that it should play a role in RSV replication. In this study, we found that cypA could interact with RSV-N, an RdRp-related protein of RSV, through its PPIase activity, so as to inhibit viral replication. This mechanism is different from other existing viewpoints on how cypA affects viral replication and may provide a new potential target to impair the RSV RdRp activity to act as a DAA for RSV infection.

## RESULTS

### cypA expression was significantly upregulated after RSV infection.

**FIG 1:**
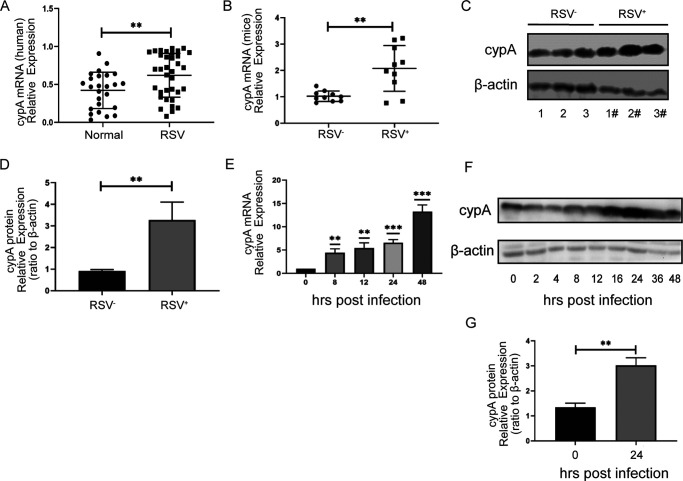
cypA expression was significantly elevated when RSV infected humans, mice, or cells. (A) The results of qRT-PCR analysis of the cypA mRNA expression in clinical samples. (B) qRT-PCR analysis of the cypA mRNA expression in lung from mice infected by RSV for 3 days. (C and D) WB analysis of the cypA protein expression in lung from mice infected by RSV for 3 days (C). cypA protein levels were quantitated by densitometry and normalized to β-actin (D). (E) qRT-PCR analysis of the cypA mRNA expression in Hep2 cells infected by RSV for the indicated time. (F and G) WB analysis of the cypA protein expression in Hep2 cells infected by RSV for the indicated time (F). cypA protein levels at 24 h postinfection were quantitated by densitometry and normalized to β-actin (G). Data are means ± SD for three independent experiments. **, *P* < 0.01; ***, *P* < 0.001.

In order to investigate cypA expression after RSV infection, first, we collected sputum specimens from 35 RSV-infected patients and 25 noninfected patients; reverse transcription-quantitative PCR (qRT-PCR) was performed to determine the cypA mRNA level, and the results showed that it was higher in RSV-infected patients than in noninfected patients (Fig. 1A). Second, we utilized qRT-PCR and Western blotting (WB) methods to examine the cypA mRNA and protein levels, respectively, in RSV-green fluorescent protein (GFP)-infected BALB/c mice compared to those in noninfected mice. The consequence was in accordance with the clinical results: either cypA mRNA or protein level was increased in the RSV-GFP-infected group compared to the control group (Fig. 1B to D). Finally, cypA mRNA and protein levels in RSV-GFP-infected Hep2 cells were detected by qRT-PCR and WB methods. The results showed that cypA mRNA and protein levels gradually increased following RSV infection (Fig. 1E to G). Based on the above-described results, we could draw the conclusion that RSV infection accelerates cypA mRNA and protein expression.

### cypA could restrict RSV replication in Hep2 cells.

**FIG 2:**
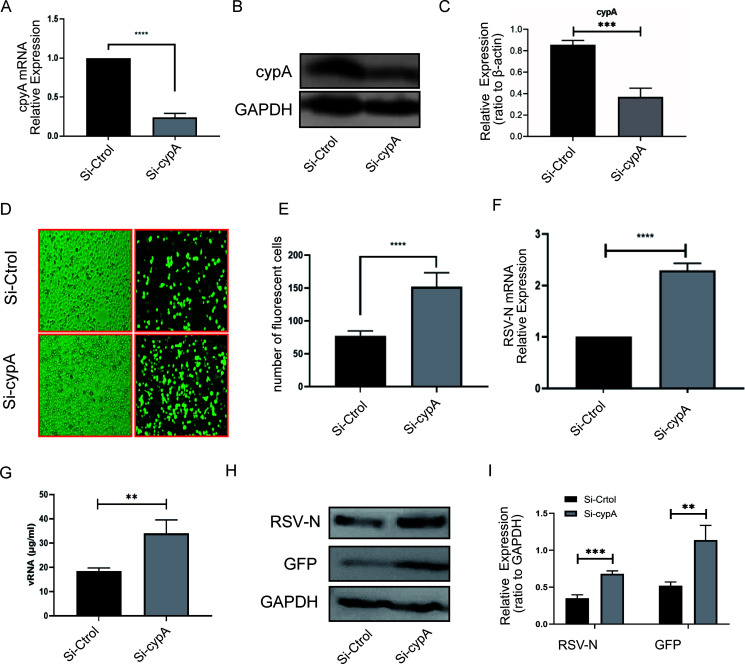
RSV replication was elevated in knockdown cypA cells. (A to C) qRT-PCR (A) and WB (B) methods were adopted to analyze the cypA mRNA or protein expression level to identify the interference efficiency of three Si-cypAs compared to Si-Ctrol. cypA protein level was quantitated by densitometry and normalized to GAPDH (C). (D and E) Fluorescence images of RSV-GFP replication in Hep2 cells transfected by Si-Ctrol or Si-cypA (D). The statistical results for the number of green fluorescent cells among Hep2 cells from 10 random microscope fields are shown (E). (F and G) qRT-PCR analysis of the RSV-N mRNA level in Hep2 cells (F) and concentration of viral RNA extracted from cell culture supernatant of Hep2 cells (G). (H and I) WB analysis of the RSV-N or GFP protein level in Hep2 cells (H). RSV-N and GFP protein levels were quantitated by densitometry and normalized to GAPDH (I). Data are means ± SD for three independent experiments. **, *P* < 0.01; ***, *P* < 0.001.

**FIG 3:**
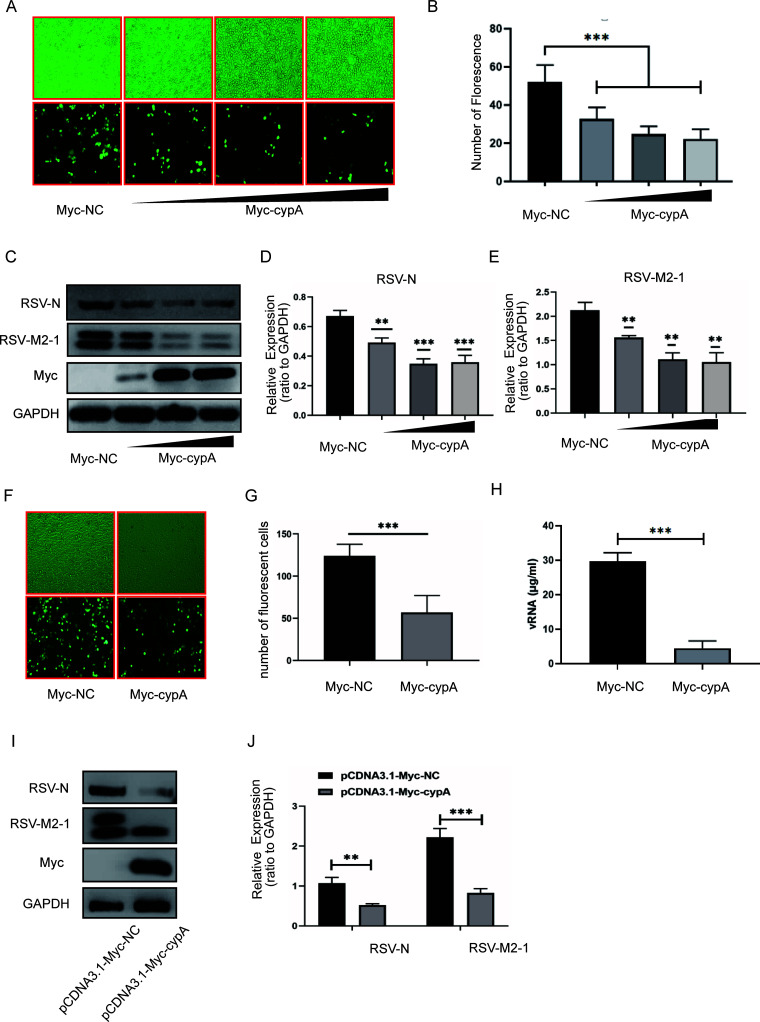
cypA could inhibit RSV replication. (A and B) Fluorescence images of RSV-GFP replication in Hep2 cells transfected with different doses of pCDNA3.1-Myc-cypA plasmid (A) and the statistical results for the number of green fluorescent cells from 10 random microscope fields for Hep2 cells transfected with plasmid as described above (B). (C to E) WB analysis of the RSV-N and RSV-M2-1 protein levels in Hep2 cells transfected with plasmid as described above (C). RSV-N (D) and RSV-M2-1 (E) protein levels were quantitated by densitometry and normalized to GAPDH. (F to H) Fluorescence images of RSV-GFP replication in A549 cells transfected with 500 ng of pCDNA3.1-Myc-cypA or 500 ng of pCDNA3.1-Myc-NC plasmid (F) and the statistical results for the number of the green fluorescent cells from 10 random microscope fields for A549 cells transfected with plasmid as described above (G). (H) Concentration of viral RNA extracted from cell culture supernatant of A549 cells. (I and J) WB analysis of the RSV-N and RSV-M2-1 protein levels in A549 cells transfected with plasmid (I). RSV-N and RSV-M2-1 protein levels were quantitated by densitometry and normalized to GAPDH (J). Data are means ± SD for three independent experiments. **, *P* < 0.01; ***, *P < *0.001.

In order to explore whether cypA plays a positive or negative role in RSV replication, we first used small interfering RNA (siRNA) technology to knock down the expression of cypA in Hep2 cells. The efficiency of knockdown was verified by qRT-PCR (Fig. 2A) and WB (Fig. 2B and C). Hep2 cells transfected with Si-cypA or Si-Ctrol for 24 h were infected with RSV-GFP (multiplicity of infection [MOI] = 1) for another 24 h; we noticed that there was more expression of green fluorescent protein (Fig. 2D and E) in the Si-cypA group than in the Si-Ctrol group. In addition, increased RSV-N mRNA level (Fig. 2F), amount of viral RNA (vRNA) extracted from cell supernatant (Fig. 2G), and RSV-N and GFP protein levels (Fig. 2H and I) were detected in the Si-cypA group, reflecting faster RSV replication. Then we transfected different doses of pCDNA3.1-Myc-cypA plasmid into Hep2 cells to increase the level of cypA protein and study whether cypA could reduce viral replication after RSV infection. Hep2 cells were infected with RSV-GFP 24 h after transfection (MOI = 1). After another 24 h, we observed the cells by fluorescence microscopy and randomly took 10 photos in each group to calculate the number of GFP-expressing cells, which reflected virus replication. The results showed that the number of GFP-expressing cells decreased significantly in a Myc-cypA-dependent manner (Fig. 3A and B). In addition, the RSV-N and RSV-M2-1 protein levels decreased accompanying the increased overexpression of cypA (Fig. 3C to E). Next, we repeated the experiment by transfecting 500 ng of pCDNA3.1-Myc-cypA or pCDNA3.1-Myc-NC into A549 cells, and the results showed that no matter the GFP number in infected cells (Fig. 3F and G), the amount of vRNA extracted from cell supernatant (Fig. 3H) and RSV-N and RSV-M2-1 protein levels (Fig. 3I and J) were consistent with those in Hep2 cells. To sum up, it is not difficult to draw the conclusion that cypA could weaken the replication of RSV.

### RSV replication was enhanced in cypA knockdown mice.

**FIG 4:**
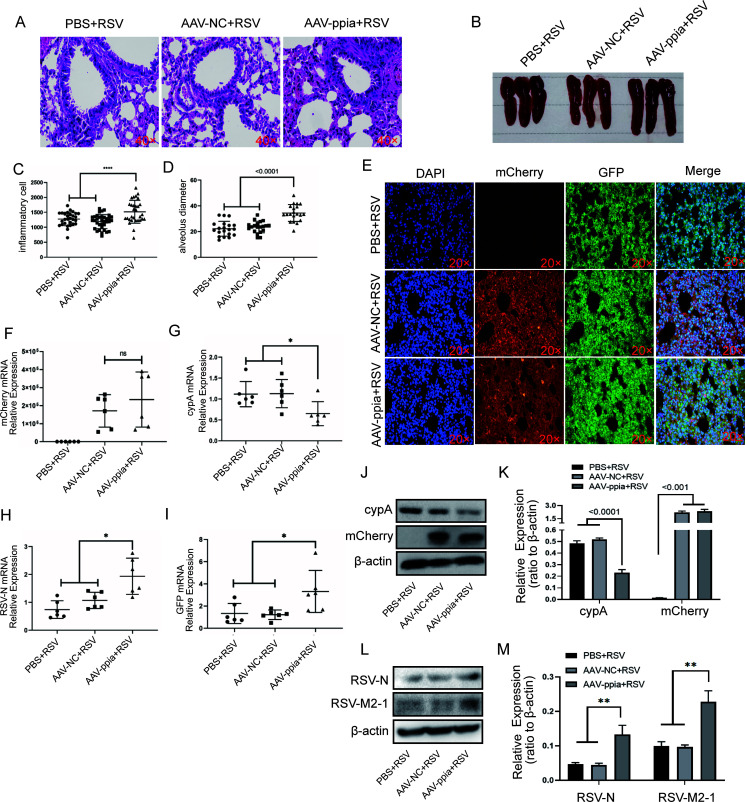
RSV replication was promoted in cypA knockdown BALB/c mice mediated by AAV-mCherry-ppia. (A) H&E results for the lung from three different groups of BALB/c mice infected by RSV for 3 days, after treatment with PBS, AAV-mCherry-NC, and AAV-mCherry-ppia, respectively, for 14 days (*n* = 6). (B) The spleens from three different groups of BALB/c mice as described above. (C and D) The statistical results for the number of inflammatory cells (C) and the diameter of alveoli (D) in H&E stain smears of three different groups of BALB/c mice as described above. (E) IF results of the lung of the BALB/c mice as described above. (F to I) The mCherry (F), cypA (G), RSV-N (H), and GFP (I) mRNA relative expressions in BALB/c mice as described above. (J and K) WB analysis of the cypA and the mCherry protein expression in three different groups of BALB/c mice as described above (J). cypA and mCherry protein levels were quantitated by densitometry and normalized to β-actin (K). (L and M) WB analysis of RSV-N and RSV-M2-1 protein in BALB/c mice as described above (L). RSV-N and RSV-M2-1protein levels were quantitated by densitometry and normalized to β-actin. Data are means ± SD. *, *P* < 0.05; **, *P* < 0.01.

To further certify the role of cypA in RSV replication, we constructed BALB/c mice with local cypA knockdown in the lung by infecting them with AAV-mCherry-ppia by nasal drip; AAV-mCherry-NC-infected and phosphate-buffered saline (PBS)-treated mice were used as controls. Two weeks after infection, the mice were infected with 10^6^ RSV virions through nasal drops for 3 days. The infection rate of AAV was evaluated by histochemical immunofluorescence (HIF) to observe the mCherry protein in lung tissue (Fig. 4E) and by qRT-PCR or WB to detect the mRNA or protein of mCherry (Fig. 4F, J, and K). At the same time, qRT-PCR and WB were used to detect the mRNA (Fig. 4G) and protein levels of cypA, respectively (Fig. 4J and K), so as to identify the knockdown efficiency of cypA in BALB/c mouse lungs. Hematoxylin and eosin (H&E) stains showed that there was more serious inflammation in the AAV-mCherry-ppia group than the AAV-mCherry-NC and PBS groups (Fig. 4A). The number of inflammatory cells (Fig. 4C) and diameter of alveoli (Fig. 4D) (we randomly selected 20 microscope fields to reflect the severity of the inflammation) were also consistent with the more severe inflammation. Besides lung inflammation, we also observed that the spleens in the AAV-mCherry-ppia group were larger than in the AAV-mCherry-NC and PBS groups (Fig. 4B). In addition, the higher GFP fluorescence intensity in HIF (Fig. 4E) and significantly increased RSV-N and GFP mRNA levels (Fig. 4H and I) and RSV-N and RSV-M2-1 protein levels (Fig. 4L and M) in the AAV-mCherry-ppia group compared with those in the AAV-mCherry-NC and PBS groups confirmed the faster replication rate of RSV. These results once again suggest that the lack of ppia could promote RSV replication.

### cypA limited RSV replication via its PPIase activity.

**FIG 5:**
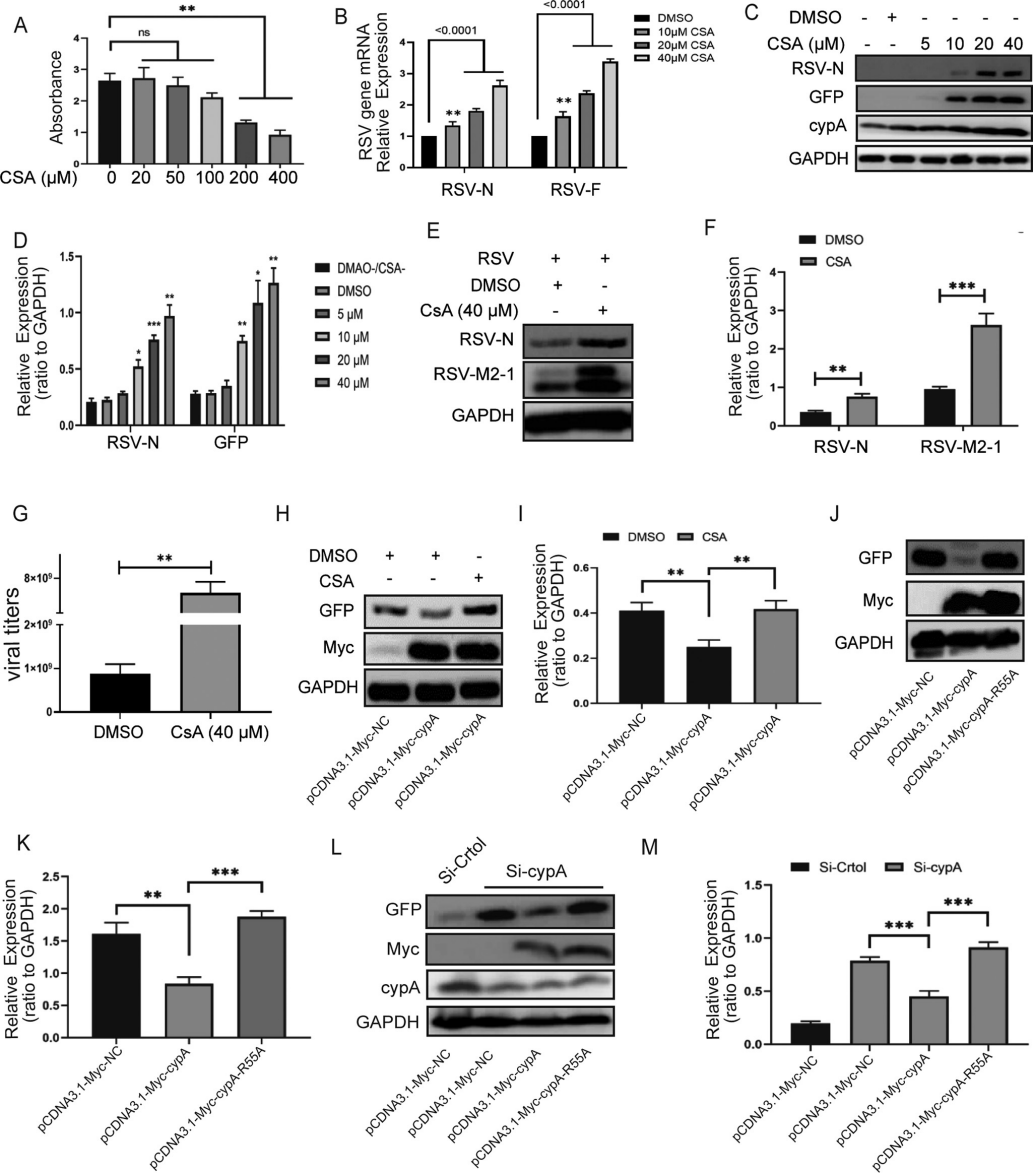
cypA inhibits RSV replication via its PPIase activity. (A) Toxicity analysis of CSA on Hep2 cells measured by CCK8. (B) The results of qRT-PCR analysis of the RSV-N and RSV-F mRNA level in Hep2 cells treated with different concentration of CSA. (C and D) WB analysis of RSV-N, GFP, and cypA protein levels in Hep2 cells treated with different concentrations of CSA (24 h) (C). RSV-N and GFP protein levels were quantitated by densitometry and normalized to GAPDH (D). (E to G) WB analysis of RSV-N and RSV-M2-1 protein levels in A549 cells treated with 40 μM CSA (48 h) (E). RSV-N and RSV-M2-1 protein levels were quantitated by densitometry and normalized to GAPDH (F). (G) Virus titer in cell culture supernatant of above A549 cells. (H and I) WB analysis of GFP protein level in Hep2 cells, which were transfected with 500 ng of pCDNA3.1-Myc-NC or 500 ng of pCDNA3.1-Myc-cypA plasmid and treated with CSA (40 μM) or DMSO (H). GFP protein level was quantitated by densitometry and normalized to GAPDH (I). (J and K) WB analysis of GFP protein level in Hep2 cells, which were transfected with 500 ng of pCDNA3.1-Myc-NC, 500 ng of pCDNA3.1-Myc-cypA, and 500 ng of pCDNA3.1-Myc-cypA-R55A plasmid (J). GFP protein level was quantitated by densitometry and normalized to GAPDH (K). (L and M) WB analysis of GFP protein level in cypA knockdown Hep2 cells, which were transfected with 500 ng of pCDNA3.1-Myc-NC, 500 ng of pCDNA3.1-Myc-cypA, and 500 ng of pCDNA3.1-Myc-cypA-R55A plasmid (L). GFP protein level were quantitated by densitometry and normalized to GAPDH (M). Data are means ± SD for three independent experiments. **, *P < *0.01; ***, *P < *0.001.

It is well known that CSA, an immunosuppressive drug, can block the activity of cypA PPIase (28). At first, the toxicity of CSA on Hep2 cells was studied with a cell proliferation kit (CCK8). The results demonstrated that there was no difference between the toxicities of CSA at 0 and 50 μM (Fig. 5A). Then we treated Hep2 cells with different concentrations of CSA for 6 h before RSV infection and carried out qRT-PCR and WB experiments 24 h after infection in order to detect mRNA levels and protein levels of RSV as measures of viral replication. The results showed that RSV-N and RSV-F mRNA levels or RSV-N and GFP protein levels were improving following the CSA concentration increase (Fig. 5B to D). The increase of cypA protein level may have been mainly caused by RSV infection because CSA has no effect on its expression (data not shown). Similar results for CSA (40 μM) enhancement RSV replication were obtained by counting the GFP (data not shown), detecting the protein levels of RSV-N and RSV-M2-1 in A549 cells by WB, and measuring the viral titers from supernatants of A549 cells (Fig. 5E to G). To confirm that CSA mainly affects the replication of RSV by blocking the activity of cypA PPIase, CSA or dimethyl sulfoxide (DMSO) was added into Hep2 cells transfected with pCDNA3.1-Myc-cypA or pCDNA3.1-Myc-NC before RSV infection. Then, after counting the GFP (data not shown), we collected cell lysate to detect GFP protein as a measure of RSV replication, and the inhibitory effect of overexpressed cypA on RSV-GFP replication was not apparent in CSA group (Fig. 5H and I). This phenomenon makes us more convinced that cypA weakens the replication of RSV through its PPIase activity. To further verify this view, we constructed another plasmid, pCDNA3.1-Myc-cypA-R55A, in which cypA arginine 55 was mutated into alanine, which resulted in a decrease of PPIase activity by nearly 99.9% (29). Not surprisingly, compared to cells transfected with control plasmid, wild-type cypA could significantly reduce the number of GFP (data not shown) and the level of GFP protein, but cypA-R55A could not (Fig. 5J and K). Similar results were obtained in cypA knockdown cells. Wild-type cypA could reduce the GFP numbers (data not shown) and the GFP protein level, but cypA-R55A could not (Fig. 5L and M). Therefore, we could conclude that cypA can inhibit the replication of RSV through its PPIase activity.

### cypA restricts RSV replication through interaction with RSV RdRp-related proteins, mainly nucleoprotein.

**FIG 6:**
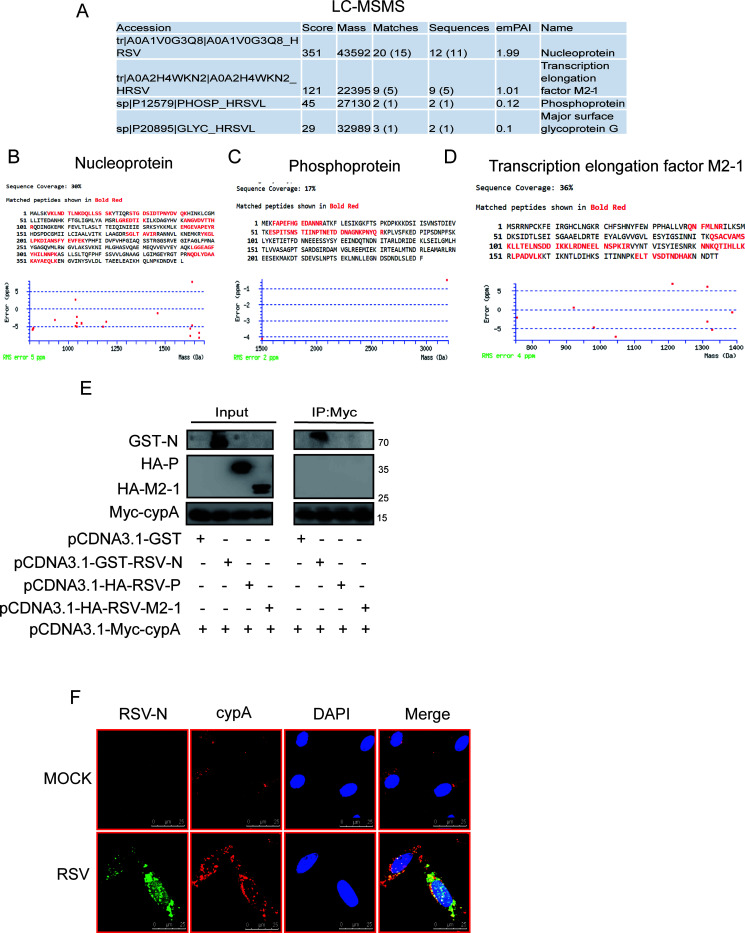
cypA could interact with RSV-N protein. (A to D) The results of LC-MS/MS showed that cypA could interact with RSV RdRp-related proteins (A). The sequence coverage of matched peptides and protein peptide mass tolerance of nucleoprotein (B), phosphoprotein (C), and transcription elongation factor M2-1 (D) in LC-MS/MS is shown. (E) Co-IP was performed after the indicated two plasmids were cotransfected into HEK293T cells for 48 h and then WB was done to identify the interaction between cypA and RSV-N, RSV-P, or RSV-M2-1. (F) Identification of the interaction between cypA and RSV-N by confocal microscopy in HEK293T cells infected by RSV.

After that, we want to know how cypA restricts RSV replication. Considering that cypA mainly affects viral replication by regulating the expression of interferon or directly binding viral proteins. Wei Liu and his colleagues had confirmed that cypA played a key and positive role in antiviral immune responses (30); we have also confirmed that cypA could increase MAVS expression (data not shown) and may increase interferon in another way (data not shown). When we blocked the interferon receptor, overexpressed cypA still showed an inhibitory effect on the replication of RSV (data not shown). Given this, we speculate that cypA could also affect RSV viral replication by other means, for example, by directly interacting with viral proteins. Therefore, in order to confirm that cypA should directly interact with RSV proteins, we performed immunoprecipitation with cypA antibodies from RSV-infected Hep2 cells and measured the supernatant by liquid chromatography-tandem mass spectrometry (LC-MS/MS). The results showed that cypA could interact with several RSV proteins, including nucleoprotein, phosphoprotein, and transcription elongation factor M2-1 (Fig. 6A). The sequence coverage rates of nucleoprotein, phosphoprotein, and M2-1 were 30%, 17%, and 36%, respectively, and the nucleoprotein-matched peptides were better than the others (Fig. 6B to D). Then we constructed pCDNA3.1-GST-RSV-N, pCDNA3.1-HA-RSV-P, and pCDNA3.1-HA-M2-1, respectively, and cotransfected them with pCDNA3.1-Myc-cypA into HEK293T cells. After cotransfection 24 h, coimmunoprecipitation (co-IP) was performed to identify the potential interaction between the proteins. The results showed the interaction existed only between Myc-cypA and glutathione *S*-transferase (GST)-RSV-N (Fig. 6E) (RSV-N was fused with GST to augment the nucleoprotein molecule to avoid overlapping with the heavy chain, and cypA could not interact with GST). Furthermore, we confirmed that cypA could combine with RSV-N by immunofluorescence (Fig. 6F).

### cypA interacted with RSV-N via its PPIase activity.

**FIG 7:**
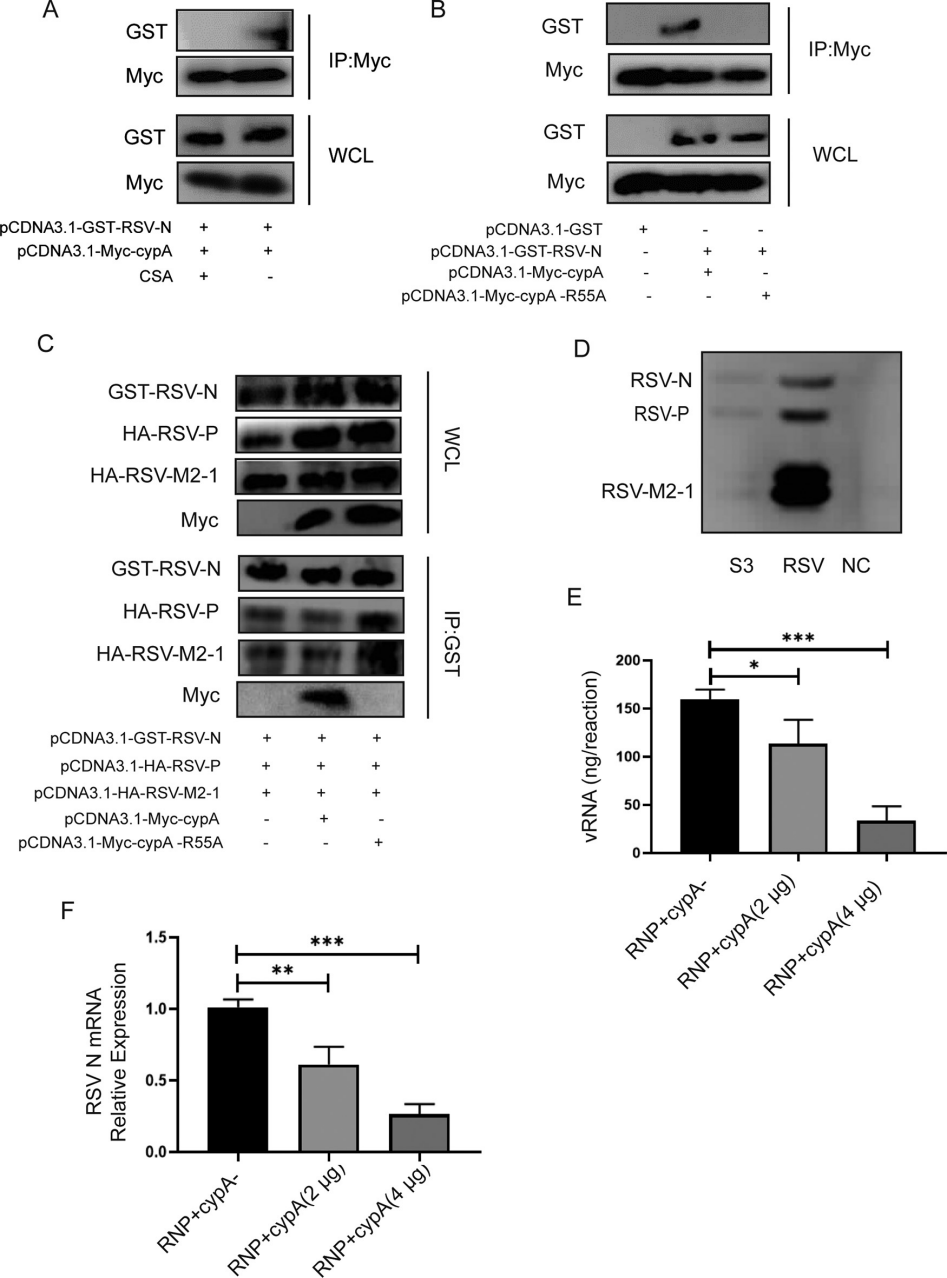
cypA interacts with RSV-N via its PPIase activity. (A) Co-IP analysis of the interaction between Myc-cypA and GST-RSV-N in cotransfected HEK293T cells treated or not with CSA. (B) Co-IP analysis of the interaction between cypA, cypA-R55A, and RSV-N in cotransfected HEK293T cells. (C) cypA could weaken the interaction between RSV RdRp-related proteins, such as RSV-N, RSV-P, and RSV-M2-1. (D) Analysis of RSV RNP in S3 fraction from RSV-infected Hep2 cells, taking total protein from either RSV-infected and mock-infected Hep2 cells as positive and negative controls. (E) Total RNA in the transcription reaction performed *in vitro*. (F) RSV N mRNA relative expression amplified by RNP with or without cypA protein. Data are means ± SD for three independent experiments. *, *P* < 0.05; ***, *P* < 0.001.

Since there was an interaction between cypA and RSV-N, we wanted to know whether this interaction depends on cypA PPIase activity. First, we treated cells with CSA for 6 h or left them untreated after cotransfection of Myc-cypA and GST-RSV-N; another 24 h later, co-IP was performed to identify if there was still interaction between them. The results demonstrated that CSA could block this interaction (Fig. 7A). Second, Myc-cypA or Myc-cypA-R55A was cotransfected with GST-RSV-N into HEK293T cells, and we did co-IP to detect their interaction. We found that there was a similar consequence as to interaction between cypA and RSV-N blocking by CSA; cypA-R55A could not interact with GST-RSV-N, either (Fig. 7B). Because RSV-N, RSV-P, and RSV-M2-1 are important components of RSV RdRp, we considered that binding of cypA to RSV-N may attenuate binding of RSV-N to RSV-P or RSV-M2-1 to affect the activity of viral RdRp. Therefore, we cotransfected HEK293T cells with three RSV plasmids and Myc-cypA or Myc-cypA-R55A plasmid. Co-IP results showed that cypA could indeed reduce the binding between RSV-N and RSV-P (Fig. 7C). After that, we extracted the RNP from RSV-infected Hep2 cells (Fig. 7D) and simulated the RNA synthesis function of RdRp *in vitro*. When cypA was added to the transcription system, the amount of RNA synthesis production was significantly reduced (Fig. 7E), and the RSV-N mRNA amplified from the isovolumic production was decreased (Fig. 7F). All these results led us to conclude that cypA interacted with RSV-N, depending on its PPIase activity to impair the function of RSV RdRp.

## DISCUSSION

Over the past decade, researchers have found that cypA could affect the replication of a variety of viruses, including DNA viruses such as hepatitis B virus (HBV) (31), human cytomegalovirus (HCMV) (32, 33), and Epstein-Barr virus (EBV) (13) and RNA viruses such as human immunodeficiency virus type 1 (HIV-1) (34–38) and flaviviruses (39). Moreover, the influence of cypA on viruses is ever changing and irregular from virus to virus. For instance, cypA enhances HBV, EBV, HIV, and HCV replication, while it reduces IAV and rotavirus (RV) replication (40). In addition, cypA relies on its PPIase activity to achieve various physiological functions. However, many studies have shown that the cypA PPIase activity is not necessary for virus replication. In our research, we found that after RSV infection, the expression of cypA was significantly increased in clinical RSV infectious patients, RSV-infected mice and Hep2 cells. And in turn, we evaluated the role of cypA in RSV replication in cypA knockdown mice and Hep2 cells and found that the lack of cypA can obviously increase the production of RSV. Moreover, cypA playing an inhibitory role in a cypA-dependent manner was confirmed by transfecting different doses of pCNDA3.1-Myc-cypA into Hep2 cells. Furthermore, we treated the cells with CSA, an inhibitor of cypA PPIase activity, or transfected them with PPIase activity-deficient plasmid pCNDA3.1-Myc-cypA-R55A, to determine whether cypA-restricted RSV replication depends on its PPIase activity. In addition, cypA can restore the inhibitory effect of viral replication in cypA knockdown cells, but cypA-R55A cannot. These results indicate that cypA suppressed RSV proliferation through its PPIase activity.

The mechanisms of cypA affecting viral proliferation and the key points of cypA targets in the viral replication cycle are also diverse among different viruses. For example, enterovirus 71 (EV71) capsid protein VP1 has an H-I loop-like structure on its surface, where cypA could bind to EV71 (12). Once combined, cypA relies on its PPIase activity to catalyze the *cis*-*trans* isomerization of proline in VP1 protein, which enables the virus to complete its uncoating (12). If serine at position 243 of VP1 protein were replaced with proline, the affinity between cypA and VP1 protein would increase significantly, while the affinity between cypA-H126Q (lack of PPIase activity) and VP1 protein would decrease significantly, which indicates that the PPIase activity of cypA is necessary for EV71 replication. cypA plays a more complicated role in the process of HIV infection. The HIV capsid protein (CA) is rich in proline at amino acid positions 85 to 90, where Gly89 and Pro90 are the binding sites for cypA (34). cypA catalyzes and isomerizes the peptide bond at Gly89/Pro90 of CA and participates in the virus replication process as a molecular chaperone, which can recognize heparin on target cells to initiate the stage of virus recognition and adsorption (35, 36). More interestingly, when the virus enters the host cell, cypA can also trigger the reverse transcription of the activated virus (37). In addition, cypA could bind to CA and integrate into the virosome. If blocking the shell formation of cypA on virus particles, the virosome packaged by host cells will not be infectious (38). Therefore, the combination of cypA and CA is essential to both HIV virosomal integrity and infectivity. However, cypA exhibits a unique mechanism in influenza virus replication that differs from what is described above. The research of Xiaoling Liu et al. has illustrated that not only cypA but also cypA-R55A could bind to M1 protein of influenza virus, thus inhibiting virus replication (41). Notably, cypA not only did not affect the replication and transcription of the viral genome but also did not block the release of mRNA from the nucleus to the cytoplasm, but it significantly reduced the expression of M1 protein (42). Further studies show that cypA played a role in blocking the binding of AIP4 to M1 protein, which leads to ubiquitination of lysine at positions 102 and 104 of K48-linked M1 protein, thus reducing the ubiquitination level of M1 and further playing a limited role in virus replication (43). In our study, we collected the supernatant of immunoprecipitation performed with cypA antibodies to detect whether there were RSV proteins in the substances of immune complex by LC-MS/MS. The results revealed that there may be interactions between cypA and RSV proteins, such as RSV-N, RSV-P, and RSV-M2-1. Finally, we confirmed that cypA combined with RSV-N in HEK293T cells with or without cotransfection with pCDNA3.1-Myc-cypA and pCDNA3.1-GST-RSV-N through immunoprecipitation and immunofluorescence. The interaction between cypA and RSV-N was blocked when we treated the cells with CSA, and cypA-R55A could not combine RSV-N. Those results demonstrated that cypA interacted with RSV-N via its PPIase activity. Therefore, will the combination of cypA and RSV-N affect the RdRp polymerase activity of RSV? We cotransfected the three plasmids of RSV-N, RSV-P, and RSV-M2-1 with Myc-cypA or Myc-cypA-R55A into HEK293T cells and found that cypA weakened the binding of RSV-N to RSV-P. In addition, we extracted RNP of RSV and simulated a transcription test *in vitro*, which showed that cypA significantly reduced the production of transcript. These results led to the preliminary conclusion that cypA weakens the activity of RSV RdRp polymerase, which is a potential target for drug design.

On the other hand, cypA inhibits or promotes virus replication by enhancing or weakening the host’s antiviral mechanism. Take RV as an example: in the early stage of RV infection, virus nonstructural protein NSP1 can activate the phosphatidylinositol 3-kinase (PI3K/Akt) signaling pathway (44), which can significantly increase the expression level of hypoxia-inducible factor 1α (HIF-1α) in cells (45). HIF-1α, which combines with the promoter of cypA (46), can strikingly increase cypA expression. Then, cypA is recruited to the viroplasm, where it binds to RV structural protein VP2 to inhibit viral replication (40). In addition, Haiyang He et al. also reported that cypA can also inhibit RV replication by promoting host cells to produce more interferon beta (IFN-β) (47) through overexpression and short hairpin RNA (shRNA). Wei Liu and his colleagues confirmed the cypA is the key positive regulator of antiviral immune responses mediated by RIG-I (30). In our study, we also explored the influence of RSV on HIF-1α expression. We observed that RSV infection indeed increased HIF-1α expression in Hep2 cells (data not shown) and that the PI3K/Akt signaling pathway was also activated after RSV infection (data not shown). When we used the catalyst or inhibitor of HIF-1α, the expression of cypA increased or decreased correspondingly, and the replication of RSV decreases or increases correspondingly (data not shown). However, which components of RSV stimulate host cells to trigger this reaction is still under further exploration. As to whether and how the high expression of cypA caused by RSV infection can promote the expression of interferon is being studied in other subjects related to the glycometabolism pathway. Conversely, interferon can also increase the expression level of cypA protein (data not shown), indicating that there is a positive regulatory relationship between cypA and interferon. In addition, when we treated cypA-overexpressing cells with interferon receptor inhibitors, RSV replication did not recover (data not shown). This indicated that the inhibition of RSV replication by cypA did not depend entirely on its interference promotion. Therefore, this study focused on the interaction between cypA and RSV-related RdRp proteins during RSV replication, which is different from other studies. We paid more attention to the influence of cypA on virus polymerase activity. Of course, the specific action sites need to be further studied in future experiments.

**FIG 8:**
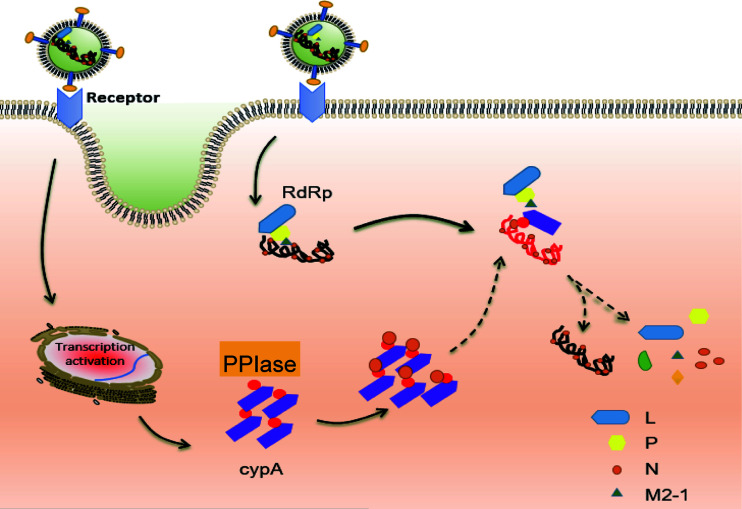
A schematic model of cypA inhibiting RSV replication through binding to RSV-N.

In conclusion, we describe a new mechanism, that is, cypA, inhibits RSV replication in host cells through its PPIase activity interacting with RSV nucleocapsid protein N, which is recognized by RSV polymerase as the most important component of RNP for initiating RSV transcription (Fig. 8). The interaction of cypA with viral polymerase can be found also in other viruses, such as HCV, vesicular stomatitis virus (VSV), and CoV. However, the interaction has different effect on the virus replication. Chatterji et al. found in their study that cypA promoted HCV replication through various mechanisms. On the one hand, cypA enhances the affinity between HCV polymerase NS5A and nonstructural protein 5B (NS5B) and viral RNA by its pocket-like PPIase activity (10). On the other hand, the cypA-NS5A complex prevents interferon regulatory factor 9 (IFR9) from binding to NS5A, thus weakening the antiviral effect of the host (48). In addition, the research of Foster’s team is basically consistent with that of Chatterji et al. They also found that cypA combined with domain II of NS5A, enhancing its ability to bind RNA, thus promoting the replication of hepatitis C virus (49). Notably, Chatterji et al. have emphasized in other studies that the mechanism of cypA regulating HCV replication is not to promote NS5A and NS5B to form a replication complex (RC) (50). VSV and RSV share similar gene structures and virus replication mechanisms. However, the research results of Bose et al. show that cypA relies on its PPIase activity to catalyze the conformational change of proline in VSV N protein, which promotes the formation of ribonucleoprotein (RNP) between N protein and the VSV genome (51) and further promotes virus replication. This is different from our research results. We speculate that the combination of cypA and RSV-N may weaken the viral polymerase activity of RSV, which needs to be confirmed by subsequent experiments.

It is particularly noteworthy that cypA promotes the replication of coronavirus. The research of Zhinan Chen et al. showed that cypA mediated the binding of HAb18G/CD147 with SARS-CoV N protein, which facilitated SARS-CoV invasion of host cells (14). Yue Ma-Lauer et al. demonstrated that there is an interaction between cyclophilin A and HCoV-229E N protein, which is inevitable for viral replication (15). In addition, C. Liu and L. Tian discussed the topic that cypA, depending on its PPIase activity, plays a positive role in SARS-CoV-2 virus replication (52, 53). But the deeper mechanism needs to be further explored.

Collectively, we illustrated a new mechanism, that cypA induced by RSV infection inhibited viral replication via its PPIase activity, which is completely different from the case with other viruses. This may provide a novel insight into RSV-host interactions and target for designing new direct-acting antiviral drugs for RSV infection.

## MATERIALS AND METHODS

### Patients.

This study was conducted in accordance with the principles of the Declaration of Helsinki. From November 2018 to January 2019, 35 clinical pediatric patients were diagnosed as RSV infected by a multiple detection kit for 13 respiratory pathogens (Health Gene Tech, Ningbo, China) in the Second Hospital of Hebei Medical University. Meanwhile, 25 noninfected volunteers without respiratory diseases served as negative controls. Sputum specimens of the inpatients and healthy volunteers were collected with a sputum aspirator based on national clinical laboratory procedures. Total RNA was collected and qualified according to the kit, and cDNA was used to perform the following experiment.

### Animals and treatment.

Four-week-old female specific-pathogen-free (SPF) BALB/c mice were purchased from the Experimental Animal Center of Hebei Medical University. All mice were housed in temperature-controlled individual ventilated cages (IVC) with 12-h light/12-h dark cycles and were fed standard chow and sterile tap water. The mice received humane care, and experiments were carried out according to the criteria outlined in the *Guide for the Care and Use of Laboratory Animals* (54) and with the approval of the Animal Care and Use Committee of Hebei Medical College.

The mice were divided into 3 groups (*n* = 6 per group). We treated the mice with PBS (20 μl) or infected them with AAV-mCherry-NC (20 μl containing about 1 × 10^11^ AAV virions, produced by GENECHEM) or AAV-mCherry-ppia (20 μl containing about 1 × 10^11^ AAV virions, produced by GENECHEM), respectively, for 2 weeks. Then we infected the 3 groups of mice with RSV-GFP (1 × 10^6^ per mouse) for 3 days. cypA and mCherry mRNA expressions in mouse lungs were detected by qPCR, and cypA and mCherry protein levels were examined by Western blotting to determine the knockdown efficiency. Viral GFP and N genes in mouse lungs were detected by qPCR, or viral protein N and M2-1 were examined by Western blotting to ascertain the RSV replication. Hematoxylin and eosin (H&E) staining and immunohistofluorescence were performed to detect the lung pathology or RSV replication in the three different groups.

### Cells and virus.

Vero cells were grown in Dulbecco’s modified Eagle’s medium supplemented with 10% fetal bovine serum (FBS) and antibiotics (penicillin and streptomycin; Solarbio) in a humidified 5% CO_2_ atmosphere at 37°C. Hep2 cells, A549 cells, and human kidney (293T) cells were grown in RPMI 1640 medium supplemented with 10% fetal bovine serum and antibiotics in a humidified 5% CO_2_ atmosphere at 37°C. RSV-GFP A strain, which was kindly provided by He Jinsheng, was amplified in Vero cells.

### Viral infection and virus titer assay.

Cells were washed with PBS once and incubated with RSV-GFP at a multiplicity of infection (MOI) of 1 or mock infected (with medium alone) for 2 h in serum-free RPMI 1640. The supernatant was aspirated after absorption, and cells were maintained with RPMI 1640 supplemented with 2% (vol/vol) FBS for the indicated times.

RSV-GFP was 10-fold diluted and used to infect Hep2 cells as described above; 3 days later, titers were detected by plaque-forming assay and expressed in PFU/milliliter of cell lysates or by counting GFP.

### RNA extraction and reverse transcription-quantitative PCR (qRT-PCR).

Total RNA was isolated with TRIzol universal RNAiso reagent (Tiangen). cDNA was generated with MonAmp ChemoHS qPCR mix (Monad). Real-time PCR was performed in an ABI Prism 7500 sequence detection system (Applied Biosystems) using PowerUp SYBR green master mix (Applied Biosystems). β-Actin was employed as an endogenous control for mRNA relative expression.

### Construction of plasmids.

Wild-type cypA and mutated cypA-R55A gene sequences were synthesized and subcloned into pCDNA3.1-Myc by GenScript Biological Technology Company (Nanjing, China). RSV-N, RSV-P, and RSV-M2-1 gene sequences, which were optimized as previously described (55), were synthesized and cloned into pCDNA3.1-GST and pCDNA3.1-HA plasmids by GenScript Biological Technology Company. All plasmids were extracted with a Qiagen Plasmid Amp minikit and split after the concentration was measured for the following experiment.

### Cell transfection.

Cells were seeded in appropriate plates according to the test requirements. Plasmids or siRNAs targeted on ppia (designed and synthesized by GenePharma) were transfected into cells with Lipofectamine 2000 (Invitrogen) following the manufacturer’s instructions.

### Immunofluorescence.

293T cells were seeded on coverslips and transiently transfected with corresponding plasmids with Lipofectamine 2000 (Invitrogen). Twenty-four hours later, the cells were washed in PBS, fixed in 4% formaldehyde for 15 min, and permeabilized with PBST (0.5% Triton X-100 in PBS) for 10 min at room temperature. The cells were washed three times with PBS and incubated in 10% normal goat serum (Solarbio) for 30 min. Primary antibodies (anti-RSV-N and anti-cypA) (ab94806; Abcam, ABclonal) were diluted with PBS containing 10% goat serum, and the cells were incubated with primary antibodies for 1 h at room temperature. After three washes, cells were incubated with secondary antibodies containing Alexa Fluor 488-labeled goat anti-rabbit (ab150080, 1:500) and Alexa Fluor 594-labeled goat anti-mouse (ab150113, 1:500). Coverslips were mounted on the glass slides with DAPI Fluoromount-G (SouthernBiotech) for fluorescence microscopy or confocal imaging.

### Immunoprecipitation and Western blot analysis.

Cell were seeded into six-well plates and maintained in medium overnight; the next day, the transient-transfection experiment was performed with corresponding plasmids for another 24 h. After careful removal of culture medium from confluent cells, the cells were washed once with PBS, 1 ml of cold PBS was added, the cells were scraped, and then the cells were shifted into a tube and centrifuged at 5,000 rpm and 4°C for 5 min, followed by addition of appropriate cell lysis buffer (Beyotime Biotechnology) for Western blot assay or lysis/wash buffer (Thermo Scientific) for immunoprecipitation with phosphate and protease inhibitors (MedChemExpress) on ice for 10 min. Cell lysates were centrifuged at 12,000 rpm and 4°C for 10 min. The supernatant was transferred into a new tube, and protein concentrations were determined with a NanoDrop 2000 spectrophotometer. The supernatant could be used to do immunoprecipitation or Western blotting. Immunoprecipitation was performed according to Pierce classic magnetic IP/co-IP kit instructions (Thermo Scientific). An equal volume of 2× sodium dodecyl sulfate polyacrylamide gel electrophoresis (SDS-PAGE) buffer was added into the supernatant and heated at 95°C for 5 min for Western blot assay. A total of 20 to 30 μg of proteins was separated by 12% SDS-PAGE, and the bands were transferred to a polyvinylidene fluoride (PVDF) membrane (Millipore), which was blocked with 5% nonfat milk. After incubation with primary antibodies specific to RSV-N (ab94806; Abcam), RSV-M2-1 (ab94805; Abcam), GFP (ABclonal), Myc (ABclonal), cypA (ABclonal), β-actin (Proteintech), or glyceraldehyde-3-phosphate dehydrogenase (GAPDH; Proteintech), the blots were incubated with horseradish peroxidase (HRP)-labeled goat anti-mouse IgG (Abgent) or HRP-labeled goat anti-rabbit IgG (Abgent) and were detected with Western Lightning plus-ECL reagent (Zeta Life) using a Synoptics Syngene bioimaging instrument. GAPDH or β-actin was used as a loading control for immunoblotting.

### vRNA extraction.

RSV was used to infect Hep2 cells after different treatments. After 3 days, cell supernatants were collected. After centrifugation, 200-μl samples were taken to extract RSV nucleic acid according to the instructions of the liquid virus RNA extraction kit (Biolab). The extracted vRNA was quantified by using Nanodrop ND-1000 (Thermo Fisher Scientific).

### RSV RNP preparation and RSV polymerase assays.

We extracted the RNP complex of RSV strictly according to the method described by Stephen W. Mason et al. and simulated the transcription of RSV polymerase *in vitro* (56). Transcription reaction mixtures (60 μl) contained 5 μl of RSV RNP (with or without cypA protein [Novoprotein]) in reaction buffer (50 mM Tris-acetate [pH 7.5], 120 mM potassium acetate, 4.5 mM MgCl_2_, 5% glycerol, 2 mM EGTA, 3 mM dithiothreitol [DTT], 50 mg/ml of bovine serum albumin [BSA], 0.4 mM [each] ATP, GTP, CTP, and UTP, and 4% dimethyl sulfoxide [DMSO]). RNA produced in the reaction system was extracted with a RNA extraction kit (Biolab) and analyzed quantitatively by NanoDrop ND-1000 (Thermo Fisher Scientific). Then the product was used to identify the RSV N mRNA by qRT-PCR.

### Statistical analysis.

Data from three independent experiments were expressed as the means ± standard deviations (SD). GraphPad Prism software was used for statistical analyses.

## References

[1] Shi T, et al. 2017. Global, regional, and national disease burden estimates of acute lower respiratory infections due to respiratory syncytial virus in young children in 2015: a systematic review and modelling study. Lancet 390:946–958. 10.1016/S0140-6736(17)30938-8. 28689664 PMC5592248

[2] Pneumonia Etiology Research for Child Health (PERCH) Study Group. 2019. Causes of severe pneumonia requiring hospital admission in children without HIV infection from Africa and Asia: the PERCH multi-country case-control study. Lancet 394:757–779. 10.1016/S0140-6736(19)30721-4. [Erratum, 394:736, 2019, 10.1016/S0140-6736(19)30721-4.] 31257127 PMC6727070

[3] Chaw PS, Hua L, Cunningham S, Campbell H, Mikolajczyk R, Nair H, RESCEU Investigators. 2020. Respiratory syncytial virus-associated acute lower respiratory infections in children with bronchopulmonary dysplasia: systematic review and meta-analysis. J Infect Dis 222(Suppl 7):S620–S627. 10.1093/infdis/jiz492. 31825072

[4] Chaw PS, Wong SWL, Cunningham S, Campbell H, Mikolajczyk R, Nair H, RESCEU Investigators. 2020. Acute lower respiratory infections associated with respiratory syncytial virus in children with underlying congenital heart disease: systematic review and meta-analysis. J Infect Dis 222(Suppl 7):S613–S619. 10.1093/infdis/jiz150. 31599958

[5] Graham BS. 2017. Vaccine development for respiratory syncytial virus. Curr Opin Virol 23:107–112. 10.1016/j.coviro.2017.03.012. 28525878 PMC5653266

[6] Ke HM, Zydowsky LD, Liu J, Walsh CT. 1991. Crystal structure of recombinant human T-cell cyclophilin A at 2.5 A resolution. Proc Natl Acad Sci U S A 88:9483–9487. 10.1073/pnas.88.21.9483. 1946361 PMC52742

[7] Nigro P, Pompilio G, Capogrossi MC. 2013. Cyclophilin A: a key player for human disease. Cell Death Dis 4:e888. 10.1038/cddis.2013.410. 24176846 PMC3920964

[8] Braaten D, Ansari H, Luban J. 1997. The hydrophobic pocket of cyclophilin is the binding site for the human immunodeficiency virus type 1 Gag polyprotein. J Virol 71:2107–2113. 10.1128/JVI.71.3.2107-2113.1997. 9032343 PMC191305

[9] Luban J, Bossolt KL, Franke EK, Kalpana GV, Goff SP. 1993. Human immunodeficiency virus type 1 Gag protein binds to cyclophilins A and B. Cell 73:1067–1078. 10.1016/0092-8674(93)90637-6. 8513493

[10] Chatterji U, Bobardt M, Selvarajah S, Yang F, Tang H, Sakamoto N, Vuagniaux G, Parkinson T, Gallay P. 2009. The isomerase active site of cyclophilin A is critical for hepatitis C virus replication. J Biol Chem 284:16998–17005. 10.1074/jbc.M109.007625. 19380579 PMC2719337

[11] Liu X, Zhao Z, Liu W. 2013. Insights into the roles of cyclophilin A during influenza virus infection. Viruses 5:182–191. 10.3390/v5010182. 23322171 PMC3564116

[12] Qing J, Wang Y, Sun Y, Huang J, Yan W, Wang J, Su D, Ni C, Li J, Rao Z, Liu L, Lou Z. 2014. Cyclophilin A associates with enterovirus-71 virus capsid and plays an essential role in viral infection as an uncoating regulator. PLoS Pathog 10:e1004422. 10.1371/journal.ppat.1004422. 25275585 PMC4183573

[13] Xin S, Du S, Liu L, Xie Y, Zuo L, Yang J, Hu J, Yue W, Zhang J, Cao P, Zhu F, Lu J. 2019. Epstein-Barr virus nuclear antigen 1 recruits cyclophilin A to facilitate the replication of viral DNA genome. Front Microbiol 10:2879. 10.3389/fmicb.2019.02879. 31921057 PMC6923202

[14] Chen Z, Mi L, Xu J, Yu J, Wang X, Jiang J, Xing J, Shang P, Qian A, Li Y, Shaw PX, Wang J, Duan S, Ding J, Fan C, Zhang Y, Yang Y, Yu X, Feng Q, Li B, Yao X, Zhang Z, Li L, Xue X, Zhu P. 2005. Function of HAb18G/CD147 in invasion of host cells by severe acute respiratory syndrome coronavirus. J Infect Dis 191:755–760. 10.1086/427811. 15688292 PMC7110046

[15] Ma-Lauer Y, Zheng Y, Malešević M, von Brunn B, Fischer G, von Brunn A. 2020. Influences of cyclosporin A and non-immunosuppressive derivatives on cellular cyclophilins and viral nucleocapsid protein during human coronavirus 229E replication. Antiviral Res 173:104620. 10.1016/j.antiviral.2019.104620. 31634494 PMC7114175

[16] Pfefferle S, Schöpf J, Kögl M, Friedel CC, Müller MA, Carbajo-Lozoya J, Stellberger T, von Dall'Armi E, Herzog P, Kallies S, Niemeyer D, Ditt V, Kuri T, Züst R, Pumpor K, Hilgenfeld R, Schwarz F, Zimmer R, Steffen I, Weber F, Thiel V, Herrler G, Thiel HJ, Schwegmann-Wessels C, Pöhlmann S, Haas J, Drosten C, von Brunn A. 2011. The SARS-coronavirus-host interactome: identification of cyclophilins as target for pan-coronavirus inhibitors. PLoS Pathog 7:e1002331. 10.1371/journal.ppat.1002331. 22046132 PMC3203193

[17] Carbajo-Lozoya J, Ma-Lauer Y, Malešević M, Theuerkorn M, Kahlert V, Prell E, von Brunn B, Muth D, Baumert TF, Drosten C, Fischer G, von Brunn A. 2014. Human coronavirus NL63 replication is cyclophilin A-dependent and inhibited by non-immunosuppressive cyclosporine A-derivatives including Alisporivir. Virus Res 184:44–53. 10.1016/j.virusres.2014.02.010. 24566223 PMC7114444

[18] Dawar FU, Tu J, Khattak MN, Mei J, Lin L. 2017. Cyclophilin A: a key factor in virus replication and potential target for anti-viral therapy. Curr Issues Mol Biol 21:1–20. 10.21775/cimb.021.001. 27033630

[19] Afonso CL, Amarasinghe GK, Bányai K, et al. 2016. Taxonomy of the order Mononegavirales: update 2016. Arch Virol 161:2351–2360. 10.1007/s00705-016-2880-1. 27216929 PMC4947412

[20] Richard CA, Rincheval V, Lassoued S, Fix J, Cardone C, Esneau C, Nekhai S, Galloux M, Rameix-Welti MA, Sizun C, Eléouët JF. 2018. RSV hijacks cellular protein phosphatase 1 to regulate M2-1 phosphorylation and viral transcription. PLoS Pathog 14:e1006920. 10.1371/journal.ppat.1006920. 29489893 PMC5847313

[21] Bakker SE, Duquerroy S, Galloux M, Loney C, Conner E, Eléouët JF, Rey FA, Bhella D. 2013. The respiratory syncytial virus nucleoprotein-RNA complex forms a left-handed helical nucleocapsid. J Gen Virol 94:1734–1738. 10.1099/vir.0.053025-0. 23677789 PMC3749527

[22] Tawar RG, Duquerroy S, Vonrhein C, Varela PF, Damier-Piolle L, Castagné N, MacLellan K, Bedouelle H, Bricogne G, Bhella D, Eléouët JF, Rey FA. 2009. Crystal structure of a nucleocapsid-like nucleoprotein-RNA complex of respiratory syncytial virus. Science 326:1279–1283. 10.1126/science.1177634. 19965480

[23] Gilman MSA, Liu C, Fung A, Behera I, Jordan P, Rigaux P, Ysebaert N, Tcherniuk S, Sourimant J, Eléouët JF, Sutto-Ortiz P, Decroly E, Roymans D, Jin Z, McLellan JS. 2019. Structure of the respiratory syncytial virus polymerase complex. Cell 179:193–204.e14. 10.1016/j.cell.2019.08.014. 31495574 PMC7111336

[24] Ouizougun-Oubari M, Pereira N, Tarus B, Galloux M, Lassoued S, Fix J, Tortorici MA, Hoos S, Baron B, England P, Desmaële D, Couvreur P, Bontems F, Rey FA, Eléouët JF, Sizun C, Slama-Schwok A, Duquerroy S. 2015. A druggable pocket at the nucleocapsid/phosphoprotein interaction site of human respiratory syncytial virus. J Virol 89:11129–11143. 10.1128/JVI.01612-15. 26246564 PMC4621127

[25] Blondot ML, Dubosclard V, Fix J, Lassoued S, Aumont-Nicaise M, Bontems F, Eléouët JF, Sizun C. 2012. Structure and functional analysis of the RNA- and viral phosphoprotein-binding domain of respiratory syncytial virus M2-1 protein. PLoS Pathog 8:e1002734. 10.1371/journal.ppat.1002734. 22675274 PMC3364950

[26] Clarke MO, Mackman R, Byun D, Hui H, Barauskas O, Birkus G, Chun BK, Doerffler E, Feng J, Karki K, Lee G, Perron M, Siegel D, Swaminathan S, Lee W. 2015. Discovery of β-D-2′-deoxy-2′-α-fluoro-4′-α-cyano-5-aza-7,9-dideaza adenosine as a potent nucleoside inhibitor of respiratory syncytial virus with excellent selectivity over mitochondrial RNA and DNA polymerases. Bioorg Med Chem Lett 25:2484–2487. 10.1016/j.bmcl.2015.04.073. 25978965

[27] Cockerill GS, Good JAD, Mathews N. 2019. State of the art in respiratory syncytial virus drug discovery and development. J Med Chem 62:3206–3227. 10.1021/acs.jmedchem.8b01361. 30411898

[28] Colgan J, Asmal M, Neagu M, Yu B, Schneidkraut J, Lee Y, Sokolskaja E, Andreotti A, Luban J. 2004. Cyclophilin A regulates TCR signal strength in CD4+ T cells via a proline-directed conformational switch in Itk. Immunity 21:189–201. 10.1016/j.immuni.2004.07.005. 15308100

[29] Zydowsky LD, Etzkorn FA, Chang HY, Ferguson SB, Stolz LA, Ho SI, Walsh CT. 1992. Active site mutants of human cyclophilin A separate peptidyl-prolyl isomerase activity from cyclosporin A binding and calcineurin inhibition. Protein Sci 1:1092–1099. 10.1002/pro.5560010903. 1338979 PMC2142182

[30] Liu W, Li J, Zheng W, Shang Y, Zhao Z, Wang S, Bi Y, Zhang S, Xu C, Duan Z, Zhang L, Wang YL, Jiang Z, Liu W, Sun L. 2017. Cyclophilin A-regulated ubiquitination is critical for RIG-I-mediated antiviral immune responses. Elife 6:e24425. 10.7554/eLife.24425. 28594325 PMC5484619

[31] Dandri M, Locarnini S. 2012. New insight in the pathobiology of hepatitis B virus infection. Gut 61(Suppl 1):i6–i17. 10.1136/gutjnl-2012-302056. 22504921

[32] Kawasaki H, Mocarski ES, Kosugi I, Tsutsui Y. 2007. Cyclosporine inhibits mouse cytomegalovirus infection via a cyclophilin-dependent pathway specifically in neural stem/progenitor cells. J Virol 81:9013–9023. 10.1128/JVI.00261-07. 17553872 PMC1951393

[33] Keyes LR, Bego MG, Soland M, St Jeor S. 2012. Cyclophilin A is required for efficient human cytomegalovirus DNA replication and reactivation. J Gen Virol 93:722–732. 10.1099/vir.0.037309-0. 22238232 PMC3542716

[34] Gamble TR, Vajdos FF, Yoo S, Worthylake DK, Houseweart M, Sundquist WI, Hill CP. 1996. Crystal structure of human cyclophilin A bound to the amino-terminal domain of HIV-1 capsid. Cell 87:1285–1294. 10.1016/s0092-8674(00)81823-1. 8980234

[35] Saphire AC, Bobardt MD, Gallay PA. 1999. Host cyclophilin A mediates HIV-1 attachment to target cells via heparans. EMBO J 18:6771–6785. 10.1093/emboj/18.23.6771. 10581250 PMC1171739

[36] Saphire AC, Bobardt MD, Gallay PA. 2000. Human immunodeficiency virus type 1 hijacks host cyclophilin A for its attachment to target cells. Immunol Res 21:211–217. 10.1385/IR:21:2-3:211. 10852119

[37] Schaller T, Ocwieja KE, Rasaiyaah J, Price AJ, Brady TL, Roth SL, Hué S, Fletcher AJ, Lee K, KewalRamani VN, Noursadeghi M, Jenner RG, James LC, Bushman FD, Towers GJ. 2011. HIV-1 capsid-cyclophilin interactions determine nuclear import pathway, integration targeting and replication efficiency. PLoS Pathog 7:e1002439. 10.1371/journal.ppat.1002439. 22174692 PMC3234246

[38] Colgan J, Yuan HE, Franke EK, Luban J. 1996. Binding of the human immunodeficiency virus type 1 Gag polyprotein to cyclophilin A is mediated by the central region of capsid and requires Gag dimerization. J Virol 70:4299–4310. 10.1128/JVI.70.7.4299-4310.1996. 8676452 PMC190362

[39] Qing M, Yang F, Zhang B, Zou G, Robida JM, Yuan Z, Tang H, Shi PY. 2009. Cyclosporine inhibits flavivirus replication through blocking the interaction between host cyclophilins and viral NS5 protein. Antimicrob Agents Chemother 53:3226–3235. 10.1128/AAC.00189-09. 19451286 PMC2715601

[40] He H, Mou Z, Li W, Fei L, Tang Y, Zhang J, Yan P, Chen Z, Yang X, Shen Z, Li J, Wu Y. 2013. Proteomic methods reveal cyclophilin A function as a host restriction factor against rotavirus infection. Proteomics 13:1121–1132. 10.1002/pmic.201100579. 23303713

[41] Liu X, Sun L, Yu M, Wang Z, Xu C, Xue Q, Zhang K, Ye X, Kitamura Y, Liu W. 2009. Cyclophilin A interacts with influenza A virus M1 protein and impairs the early stage of the viral replication. Cell Microbiol 11:730–741. 10.1111/j.1462-5822.2009.01286.x. 19207730

[42] Liu X, Zhao Z, Xu C, Sun L, Chen J, Zhang L, Liu W. 2012. Cyclophilin A restricts influenza A virus replication through degradation of the M1 protein. PLoS One 7:e31063. 10.1371/journal.pone.0031063. 22347431 PMC3275614

[43] Mahesutihan M, Zheng W, Cui L, Li Y, Jiao P, Yang W, Liu W, Li J, Fan W, Yang L, Liu W, Sun L. 2018. CypA regulates AIP4-mediated M1 ubiquitination of influenza A virus. Virol Sin 33:440–448. 10.1007/s12250-018-0058-6. 30328013 PMC6235765

[44] Bagchi P, Dutta D, Chattopadhyay S, Mukherjee A, Halder UC, Sarkar S, Kobayashi N, Komoto S, Taniguchi K, Chawla-Sarkar M. 2010. Rotavirus nonstructural protein 1 suppresses virus-induced cellular apoptosis to facilitate viral growth by activating the cell survival pathways during early stages of infection. J Virol 84:6834–6845. 10.1128/JVI.00225-10. 20392855 PMC2903281

[45] Jiao M, Nan KJ. 2012. Activation of PI3 kinase/Akt/HIF-1α pathway contributes to hypoxia-induced epithelial-mesenchymal transition and chemoresistance in hepatocellular carcinoma. Int J Oncol 40:461–468. 10.3892/ijo.2011.1197. 21922131

[46] Choi KJ, Piao YJ, Lim MJ, Kim JH, Ha J, Choe W, Kim SS. 2007. Overexpressed cyclophilin A in cancer cells renders resistance to hypoxia- and cisplatin-induced cell death. Cancer Res 67:3654–3662. 10.1158/0008-5472.CAN-06-1759. 17440077

[47] He H, Zhou D, Fan W, Fu X, Zhang J, Shen Z, Li J, Li J, Wu Y. 2012. Cyclophilin A inhibits rotavirus replication by facilitating host IFN-I production. Biochem Biophys Res Commun 422:664–669. 10.1016/j.bbrc.2012.05.050. 22609402

[48] Bobardt M, Hopkins S, Baugh J, Chatterji U, Hernandez F, Hiscott J, Sluder A, Lin K, Gallay PA. 2013. HCV NS5A and IRF9 compete for CypA binding. J Hepatol 58:16–23. 10.1016/j.jhep.2012.08.007. 22902549 PMC3527675

[49] Foster TL, Gallay P, Stonehouse NJ, Harris M. 2011. Cyclophilin A interacts with domain II of hepatitis C virus NS5A and stimulates RNA binding in an isomerase-dependent manner. J Virol 85:7460–7464. 10.1128/JVI.00393-11. 21593166 PMC3126559

[50] Chatterji U, Bobardt MD, Lim P, Gallay PA. 2010. Cyclophilin A-independent recruitment of NS5A and NS5B into hepatitis C virus replication complexes. J Gen Virol 91:1189–1193. 10.1099/vir.0.018531-0. 20107018 PMC2888154

[51] Bose S, Mathur M, Bates P, Joshi N, Banerjee AK. 2003. Requirement for cyclophilin A for the replication of vesicular stomatitis virus New Jersey serotype. J Gen Virol 84:1687–1699. 10.1099/vir.0.19074-0. 12810862

[52] Liu C, von Brunn A, Zhu D. 2020. Cyclophilin A and CD147: novel therapeutic targets for the treatment of COVID-19. Med Drug Discov 7:100056. 10.1016/j.medidd.2020.100056. 32835213 PMC7364167

[53] Tian L, Liu W, Sun L. 2020. Role of cyclophilin A during coronavirus replication and the antiviral activities of its inhibitors. Sheng Wu Gong Cheng Xue Bao 36:605–611. 10.13345/j.cjb.200049. (In Chinese.) 32347055

[54] National Research Council. 2011. Guide for the care and use of laboratory animals, 8th ed. National Academies Press, Washington, DC.

[55] Lo MS, Brazas RM, Holtzman MJ. 2005. Respiratory syncytial virus nonstructural proteins NS1 and NS2 mediate inhibition of Stat2 expression and alpha/beta interferon responsiveness. J Virol 79:9315–9319. 10.1128/JVI.79.14.9315-9319.2005. 15994826 PMC1168759

[56] Mason SW, Lawetz C, Gaudette Y, Dô F, Scouten E, Lagacé L, Simoneau B, Liuzzi M. 2004. Polyadenylation-dependent screening assay for respiratory syncytial virus RNA transcriptase activity and identification of an inhibitor. Nucleic Acids Res 32:4758–4767. 10.1093/nar/gkh809. 15356293 PMC519107

